# Supplementary material for: Social representation of fair price among professional photographers
Source: PLoS One. 2020 Dec 22;15(12):e0243547. doi: 10.1371/journal.pone.0243547 (PMC7755223; doi:10.1371/journal.pone.0243547)
Supplement: Attachment — Submitted filename: Response to reviewers.doc [file pone.0243547.s001.doc]

**Rebuttal Letter**

August 20, 2020

Dear Editors and reviewers of PLOS ONE,

First of all, thank you all for taking the time to review our manuscript. I found all the comments were very good and really help to improve the article. Also, your feedbacks were provided with very good guidance.

I sincerely tried to address the points that were raised but I am willing to work on them again if you ever think that this new version is not good enough. We were able to reduce the intro like everybody have suggested.

I would like to address some points that were raised by both reviewers.

Reviewer #1

I agree that it would be interesting to have different participants that provide handicraft artworks but this research was financed by a photography company which allow us to get access to a lot of photographers. They were mainly concerned in promoting a better relationship among the photographers that work with them. As a researcher, I was interested in exploring the concept of fair price with their sample and provide some practical guidance to them. This is something I could do when I presented the results.

In the new version of the manuscript, I tried to address why the method of social representation is an interesting method for this kind of research. It allowed us to explore freely how fair price is conceived on people that have to deal with it in their professional life.

I agree about the ambiguity this type of studies provide. It is hard to be completely sure about what these words are saying. This couId be dealt with if we had other social representation studies on the topic of fair price or among photographers. I did not find any. I preferred, and still prefer, in staying more pragmatic about what we can say so we avoid misleading the readers.

Regarding the minor issues you have addressed, I did not have more demographics questions than the ones we presented in the manuscript. I tried to provide more explanations about the factorial analysis method like you have suggested.

Reviewer #2

I really appreciated your comments and I have to say that I am new in this world of fairness and economy. I’m not sure that I understood what you meant by “correlation between fair price and words”. What a study using the social representation method does is to ask people to provide words that they associate with the inductive word. The frequency of a word is very important here because it reveals a common association between two words.

I formulated the research question as you have requested. Our main concern was to see how the photographers see fair price. The sample we had available allow us to explore this in a cross-cultural perspective.

It is hard to say if the sample we’ve got is representative but I believe it could be considered to be when we are dealing with photographers. I found it hard to generalise our results to other kinds of handicraft artwork but I think this work might be helpful.

Thank you, once again, for all your great comments. I hope you all will be satisfied with this new version. We are open to new suggestions.

With my best regards,

Dr. CF
